# Variable Knee-Joint Morphology In Valgus and Non-valgus Aligned Pediatric Patients With Anterior Cruciate Ligament Rupture

**DOI:** 10.1177/23259671251365625

**Published:** 2025-09-25

**Authors:** Alexander H. Seeto, Kylie Bradford, David Bade, Liam Johnson, Sheanna Maine, David J. Saxby, Christopher P. Carty, Ivan P. Astori

**Affiliations:** * School of Medicine and Dentistry, Griffith University, Gold Coast, Queensland, Australia; † Australian Centre for Precision Health and Technology, Griffith University, Gold Coast, Queensland, Australia; ‡ Department of Orthopaedics, Children's Health Queensland Hospital and Health Service, Brisbane, Queensland, Australia; Investigation performed at the Queensland Children's Hospital (Children’s Health Queensland Hospital and Health Service), Brisbane, Queensland, Australia

**Keywords:** adolescents, alignment, anterior cruciate ligament, children, lateral femoral condyle, pediatric, tibial slope, valgus

## Abstract

**Background:**

Lower limb valgus alignment has previously been identified in pediatric patients with anterior cruciate ligament (ACL) rupture. This study aimed to evaluate knee morphology on magnetic resonance imaging (MRI) in valgus and non-valgus aligned pediatric patients with ACL rupture and compare them against a typically developed (TD) cohort without ACL rupture.

**Hypothesis:**

There would be differences in knee morphology between valgus and non-valgus aligned pediatric patients with ACL rupture and a TD cohort.

**Study Design:**

Cross-sectional study; Level of evidence, 3.

**Methods:**

Data were extracted from the Queensland Children's Hospital prospective pediatric ACL Injury Registry. Preoperative MRI of patients with ACL rupture was compared against an age- and sex-matched TD cohort without knee pathology. The following morphological parameters were measured: lateral femoral condyle index (LFCI), lateral tibial height, medial posterior slope, lateral posterior slope (LTS), medial tibial depth, and notch width index. The mechanical axis deviation (MAD) was measured from preoperative long-leg radiographs. One-way analysis of covariance with the Tukey post-hoc test compared parameters in valgus (MAD <1 mm medial), non-valgus (MAD ≥1 mm medial), and TD groups, adjusting for age and sex. Mean differences were reported with 95% CIs.

**Results:**

A total of 150 patients with ACL rupture were eligible. After propensity score matching against the TD cohort (n = 26), the ACL rupture group (valgus, n = 26; non-valgus, n = 26) had similar baseline characteristics: combined mean age (13.97 ± 2.20 years) and sex (57.69% women vs 42.31% men). Compared with the TD cohort, valgus ACL-ruptured patients had significantly smaller LFCI (–0.16 [95% CI, –0.20 to −0.11]) and larger LTS (2.53 [95% CI, 0.50 to 4.56]) values. Valgus-aligned patients with ACL rupture had smaller LFCIs compared with non-valgus-aligned patients with ACL rupture (–0.12 [95% CI, 0.17 to −0.08]).

**Conclusion:**

Compared with a TD cohort, morphological variations were found within subsets of pediatric patients with ACL rupture when accounting for lower limb alignment. Valgus-aligned patients had smaller LFCI and steeper LTS values than the TD cohort.

Epidemiological studies have shown an increasing incidence of anterior cruciate ligament (ACL) ruptures worldwide,^14,27^ with the steepest annual rise observed in pediatric populations.^13,23-25^ This increase in incidence among children and adolescents is especially concerning, given that osteoarthritic changes to the knee may occur within a decade of ACL rupture.^11,26^ Moreover, ACL rupture is associated with a large lifetime economic burden^20^ due to operative management, rehabilitation, reinjury, and degenerative sequelae such as posttraumatic osteoarthritis. In response to this emerging burden, the International Olympic Committee released a consensus statement^1^ calling for further research into injury prevention strategies and identifying baseline risk factors in pediatric patients with ACL rupture. Although it is well established that ACL rupture is caused by aberrant tibiofemoral biomechanics that stress the ACL beyond its restraining capability, few knee joint morphological risk factors have been identified to date as predictors of ACL rupture in the pediatric population. Most notably, increased lateral posterior tibial slope has been identified as a risk factor for pediatric ACL rupture in several magnetic resonance imaging (MRI) studies.^2,4^ However, it remains to be determined whether other factors play a significant role.

Morphological asymmetry between the medial and lateral knee compartments may influence the risk of ACL rupture (and concomitant meniscal injury) due to altered joint loading and increased ACL strain. The lateral femoral condyle index (LFCI) was first described by Hodel et al^8^ and is a measure of the sphericity of the lateral femoral condyle. Smaller LFCI values have been associated with an increased risk of both primary ACL rupture and graft failure in the adult population.^8^ More recently, no significant differences in LFCI were identified in a study of adult athletes with ACL rupture when comparing their uninjured knees to a healthy control group.^17^ However, this assumes that the uninjured limb had the same bony morphology as the injured limb at the time of injury.

In the pediatric population, the relationship between LFCI and ACL rupture has yet to be evaluated, and symmetry in bony morphology between limbs cannot be assumed. A registry study of 104 pediatric patients found that the ACL ruptured lower limb was insignificantly more valgus compared with the contralateral side on preoperative long-leg alignment films.^22^ This study controlled for potential confounding of postinjury swelling observed in long-leg alignment films^7^ by allowing an extended time to imaging, further validating the finding of asymmetrical alignment. Contributors to altered coronal plane alignment in pediatric patients with ACL rupture remain to be determined; however, abnormal lateral compartment morphology may play a role. Using the Queensland Children's Hospital (QCH) Pediatric ACL Registry, this study sought to compare knee bony morphology on MRI between 3 groups: (1) valgus and (2) non-valgus aligned pediatric patients who presented with ACL rupture, and (3) a typically developed (TD) control cohort. We hypothesized that there would be differences in bony morphology between valgus and non-valgus aligned pediatric patients with ACL rupture; valgus aligned patients and the TD control cohort; and non-valgus aligned patients and the TD control cohort.

## Methods

### Participant Recruitment

Institutional ethics approval for this study was received, and the guardians of all participants provided written informed consent. The study participants were recruited prospectively from the QCH Pediatric ACL Injury Registry between September 2018 and January 2024. Patients were enrolled after outpatient orthopaedic consultations for their ACL injuries. An a priori power analysis was conducted to determine participant numbers based on mean literature values^8^ for an LFCI of 0.67 in ACL ruptured patients and an LFCI of 0.70 in uninjured patients, with a pooled standard deviation of 0.04. With an alpha of .05, a power of 0.80, and a calculated effect size of 0.75, the projected sample size required was 23 participants in each group (G*Power Version 3.1.9.7).

Included patients were aged ≤16 years and had sustained noncontact ACL injuries for which surgical management was indicated. Contact ACL injuries were excluded to maintain focus on the distinct biomechanics of noncontact injuries. Patients with additional and multiligament knee injuries were excluded to isolate morphological factors specific to ACL injuries and avoid confounding from ligamentous instability.

For comparison, the TD cohort was recruited from the local community. It consisted of individuals aged ≤16 years who were free from lower limb injuries or ongoing musculoskeletal issues. Recruitment efforts involved community outreach through schools, sports clubs, and hospital staff networks. TD participants underwent MRI for comparison purposes, but not long-leg radiographs, as there was no clinical indication for radiographic imaging in this group.

The exclusion criteria for both groups included previous ACLR, congenital limb abnormalities, bony or soft tissue pathology affecting anatomy (infection or trauma), bilateral ACL injuries, and neurological disorders.

**Figure 1.**
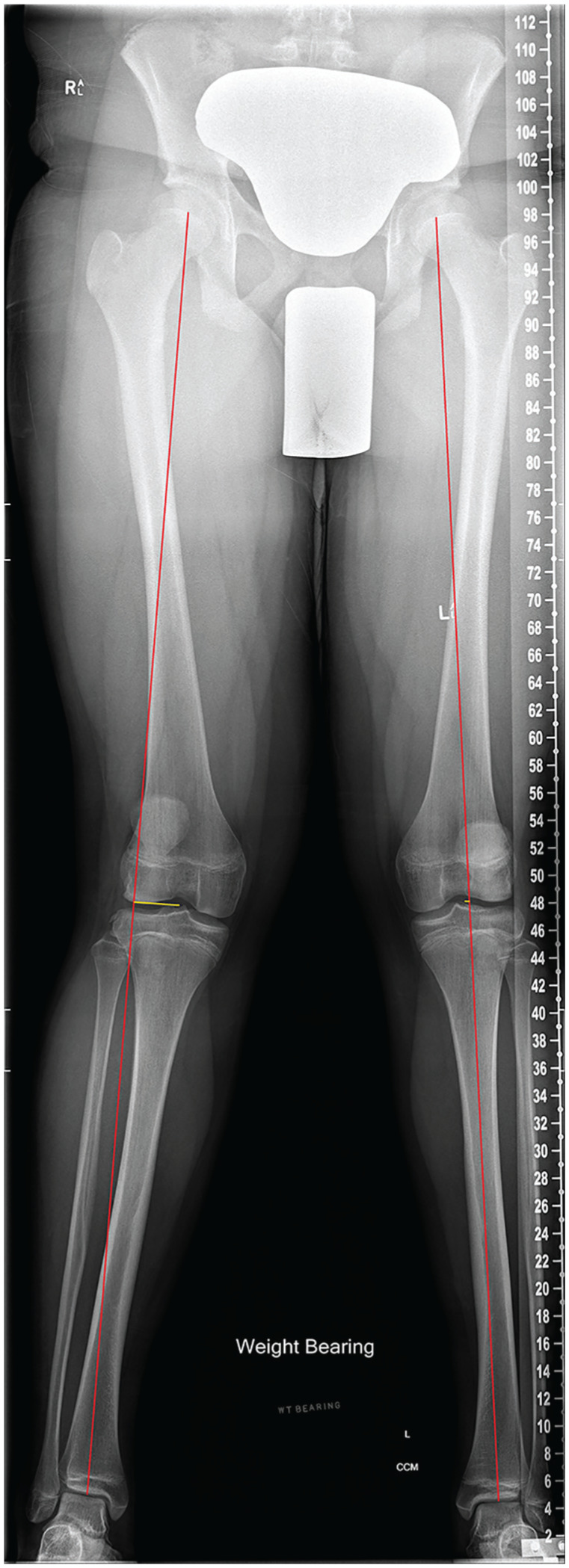
An example of MAD measurement. A participant with a right ACL rupture. MAD injured versus uninjured: 33.5 mm lateral versus 4.1 mm lateral. ACL, anterior cruciate ligament; MAD, mechanical axis deviation.

In patients with ACL rupture, baseline weightbearing long-leg alignment films were used to determine the mechanical axis deviation (MAD) (Figure 1). Long-leg radiographs were weightbearing, digitally stitched hip-knee-ankle teleoroentgenograms, standardized with forward-facing patellae and knees in full extension. Although the time to medical imaging in our registry-based cohort followed standard clinical care, our radiographic protocol enabled accurate alignment assessment. These radiographs were conducted when patients could fully extend their knees and mobilize without aids. Shorter intervals immediately after ACL injury can overestimate malalignment, while longer intervals may introduce growth-related changes or joint adaptations, such as chondral wear or condylar flattening. Our imaging protocol balances these trade-offs to ensure accurate and reliable alignment assessment. The MAD was then calculated as the perpendicular distance between the knee center and the mechanical axis, which was measured as the line joining the hip and ankle centers. The lateral MAD described a mechanical axis lateral to the knee center, whereas the medial MAD described a mechanical axis medial to the knee center. The mechanical lateral distal femoral angle (mLDFA) was measured as the angle formed by the mechanical axis of the femur and the tangent to the distal femoral joint line. The medial proximal tibial angle (MPTA) was measured as the angle formed by the mechanical axis of the tibia and the tangent to the proximal tibial joint line. These measurements allowed for a detailed evaluation of the femoral and tibial contributions to overall alignment. Based on reference ranges described by Paley et al,^19^ these patients were then divided into valgus (MAD <1 mm medial) and non-valgus subgroups (MAD ≥ 1 mm medial) for analysis.

### Measurement of Morphological Parameters

**Figure 2.**
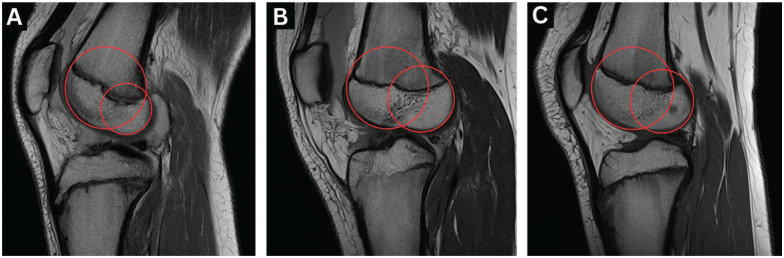
An example of LFCI measurement. (A) A patient in the valgus group, LFCI = 0.66. (B) A patient in the non-valgus group, LFCI = 0.81. (C) A patient in the TD control group, LFCI = 0.79. LFCI, lateral femoral condyle index; TD, typically developed control group.

For each participant, baseline MRI (1.5 T scanner, MAGNETOM Avantofit syngo MR VE 11-B; Siemens) of the injured limb (3D PD SPACE sequence, slice thickness: 1.0 mm, voxel size: 0.83 × 0.83 × 1.0–mm) was collected from our institution's picture archiving and communications system. The following morphological parameters were measured: LFCI^8^ (Figure 2), lateral tibial slope (LTS),^10^ lateral tibial height (LTH), medial posterior tibial slope angle (MTS),^10^ medial tibial depth (MTD), and notch width index (NWI)^3^ (Supplementary Figure S1). The LFCI was measured using the method described by Hodel et al.^8^ Briefly, the midsagittal plane of the lateral condyle was identified at the level of the popliteal groove on the coronal plane, and the corresponding sagittal slice was identified. On the sagittal slice, the extension curvature of the femoral condyle was approximated by drawing an anterior circle fitted to the subchondral bone at 6 and 9 o'clock positions. Similarly, the flexion curvature was approximated by a posterior circle fitted to the subchondral bone at the 6 and 3 o'clock positions. The LFCI was calculated as the ratio of the diameter of the flexion circle to the diameter of the extension circle. To minimize the effect of potential geometric variability or slice differences in LFCI measurements,^17^ we adopted a conservative approach aimed at avoiding the overestimation of group differences. This approach was designed to conservatively err toward the null hypothesis, reducing the risk of type 1 error and enhancing the robustness and interpretability of our findings.

The LTS was calculated according to the method outlined by Hudek et al.^10^ To establish a tibial reference line, a sagittal slice was selected where the tibial attachment of the posterior cruciate ligament, the intercondylar eminence, and the anterior and posterior tibial cortices were most concave. Two circles were drawn: (1) the cranial circle contacted the anterior, posterior, and cranial tibial cortex, and (2) the caudal circle contacted the anterior and posterior cortex, with its center point overlapping the circumference of the cranial circle. A line through the center of these circles defined the reference line. The LTS was measured by identifying the sagittal slice at the mediolateral center of the lateral tibial plateau and calculating the angle between a line perpendicular to the reference line and the tangent to the lateral plateau. The LTH was measured on the same sagittal slice. It was defined as the perpendicular distance between the subchondral bone of the lateral tibial plateau and the most proximal point of the chondral surface. The MTS was measured using the same tibial reference line described by Hudek et al.^10^ The sagittal slice at the mediolateral center of the medial plateau was identified. The angle between a line perpendicular to the reference line and the tangent to the medial plateau defined the MTS. The MTD^6^ was measured on the same sagittal slice as the MTS. It was defined as the perpendicular distance between the MTS line and the lowest point of the subchondral bone on the medial plateau. The NWI^3^ was calculated as the ratio of the width of the intercondylar notch to the width of the distal femur at the level of the popliteal groove.

Skeletal maturity was assessed for all participants using knee MRI following the methods described by Meza et al.^16^ Patients with >12 months of growth remaining were defined as women with a skeletal age of <12 years and men with a skeletal age of <15 years, corresponding to the absence of complete ossification in the tibial tubercle apophysis.

All measurements were performed by a single author (A.H.S.). To evaluate intrarater reliability, the primary measurer repeated measurements on a randomly generated subset of 15 patients 2 weeks after the initial measurement. Interrater reliability was assessed on the same randomly generated subset of 15 patients by the senior author (I.P.A.), a fellowship-trained consultant pediatric orthopaedic surgeon. Intra- and interrater reliability were reported using intra- and interclass correlation coefficients (ICCs), respectively.

### Statistical Analysis

Normality was assessed using the Shapiro-Wilk method. Continuous data were recorded as the mean and standard deviation if parametric or as the median and interquartile range (IQR) if nonparametric. Categorical variables were recorded as the frequency and percentage. Intra- and interrater reliability (2-way random with absolute agreement) were calculated using the ICC and were interpreted in accordance with the method outlined by Koo and Li.^12^

**Figure 3.**
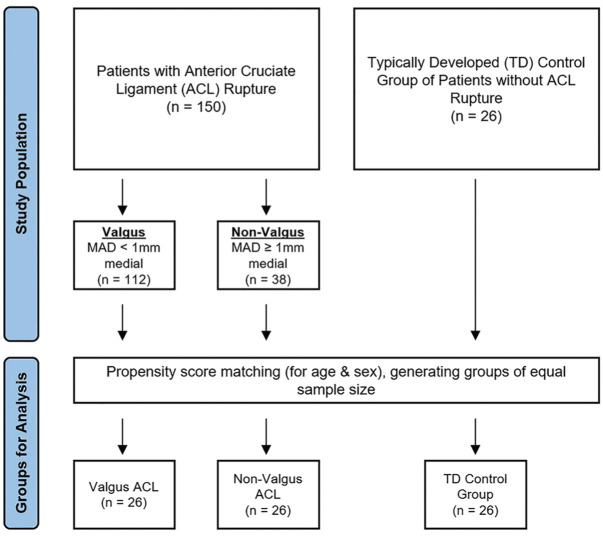
Patient grouping flow diagram. MAD, using reference ranges from Paley et al.^19^ ACL, anterior cruciate ligament; MAD, mechanical axis deviation.; TD, typically developed.

To generate groups of equal sample size, propensity score matching accounting for age and sex was performed separately for both valgus and non-valgus groups, relative to the TD control group (n = 26) (Figure 3). We evaluated the effect of group on imaging measurements with analysis of covariance (ANCOVA) using the covariates of age and sex. The Tukey post-hoc test was used to compare parameters in valgus (MAD <1 mm medial), non-valgus (MAD ≥1 mm medial), and TD groups. To validate these findings, an analysis using a more conservative alignment classification of the MAD^15^ (valgus: MAD ≥10 mm lateral and non-valgus: MAD < 10 mm lateral) was also conducted. Likewise, a supplementary ANCOVA was performed on the entire cohort before matching to demonstrate that observed effects were robust across both analyses, were not an artifact of the matching process, and were generalizable to the entire study population. Statistical analyses were performed using R Version 4.3.3 (The R Foundation) using 2-sided statistical tests with significance set at *P* < .05.

Supplementary analyses explored the relationship between age and morphological measures using correlation coefficients (Pearson or Spearman, as appropriate). Scatterplots and histograms were generated to visualize these relationships (Supplementary Table S3, Supplementary Figures S3 and S4).

## Results

There were 150 eligible patients within the QCH pediatric ACL registry, of whom 112 and 38 were categorized into valgus and non-valgus groups, respectively (Figure 3). The control group consisted of 26 TD patients. The primary analysis was conducted on a matched cohort (n = 78) of 26 patients each in the valgus, non-valgus, and TD groups. Age, sex, and body mass index were similar between valgus ACL ruptured, non-valgus ACL ruptured, and TD groups after age and sex propensity score matching into groups of equal sample size (Table 1). Approximately 30% of each cohort had >12 months of skeletal growth remaining. Because this was a registry study, the time to imaging followed standard clinical care, potentially contributing to the nonparametric distribution of the data. The median time from injury to MRI was 17 days (IQR, 5-72 days). Long-leg alignment films were performed at a median of 45 days after injury (IQR, 28-106 days). Patients undergoing long-leg radiographs were able to mobilize without aids and fully extend their injured knee, minimizing bias from acute injury-related factors such as effusion or involuntary muscle guarding. All measures showed excellent intrarater reliability and good to excellent interrater reliability (Table 2).

**Table 1:** Comparison of Baseline Characteristics Between Groups*^*a*^*

Characteristic	TD Patients (n = 26)	Valgus Patients With ACL Rupture (n=26)	Non-Valgus Patients with ACL Rupture (n = 26)
Age, years	13.37 ± 2.80	14.17 ± 2.10	14.39 ± 1.41
Male sex	11 (42.31)	11 (42.31)	11 (42.31)
BMI, kg/m^2^	21.52 ± 3.97	25.11 ± 4.46	22.46 ± 4.69
Patients with >12 months of growth remaining	10 (38.46)	8 (30.77)	9 (34.62)
Mechanical axis deviation, mm	N/A	9.23 ± 5.98 (Lateral)	10.06 ± 6.40 (Medial)
mLDFA, deg	N/A	84.40 ± 2.01	87.21 ± 2.06
MPTA, deg	N/A	88.93 ± 2.40	86.88 ± 1.94

a Data are parametric and reported as mean ± SD, or n (%). ACL, anterior cruciate ligament; BMI, body mass index; mLDFA, mechanical lateral distal femoral angle; MPTA, medial proximal tibial angle; N/A, not applicable; TD, typically developed.

**Table 2:** Intra- and Interrater Reliability of Morphological Parameters*^*a*^*

Parameter	Intrarater Reliability	Interrater Reliability
LFCI	0.95 (0.92-0.97)	0.91 (0.83-0.99)
LTS, deg	0.93 (0.90-0.95)	0.96 (0.82-0.99)
LTH, mm	0.91 (0.84-0.94)	0.91 (0.84-0.94)
MTS, deg	0.94 (0.91-0.97)	0.91 (0.84-0.98)
MTD, deg	0.90 (0.84-0.95)	0.94 (0.78-0.99)
NWI	0.92 (0.83-0.94)	0.87 (0.77-0.97)
MAD, mm	0.99 (0.99-1)	0.99 (0.98-1)
mLDFA, deg	0.96 (0.91-0.99)	0.93 (0.83-0.97)
MPTA, deg	0.98 (0.96-0.99)	0.95 (0.84-0.98)

a Data are presented as ICC (95% CI). ICC, intraclass correlation coefficient; LFCI, lateral femoral condyle index; LTH, lateral tibial height; LTS, lateral posterior tibial slope angle; MAD, mechanical axis deviation; mLDFA, mechanical lateral distal femoral angle; MPTA, medial proximal tibial angle; MTD, medial tibial depth; MTS, medial posterior tibial slope angle; NWI, notch width index.

**Figure 4.**
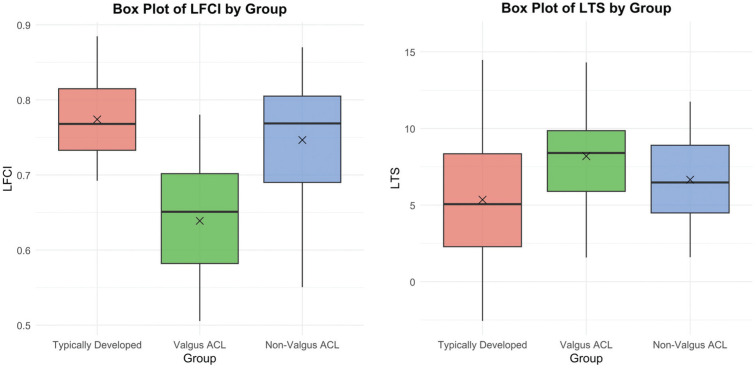
Box plots of the LFCI and the LTS. Black crosses represent group means. The valgus ACL group: MAD <1 mm medial; the non-valgus ACL group: MAD ≥1 mm medial. ACL, anterior cruciate ligament; LFCI, lateral femoral condyle index; LTS, lateral posterior tibial slope.

When comparing LFCI values to the TD reference group, valgus ACL-ruptured patients had significantly smaller LFCI values, whereas non-valgus ACL-ruptured patients showed no significant difference (Table 3). There were significant mean differences when comparing the LFCI in the non-valgus ACL group with the valgus ACL group, where patients with valgus alignment had smaller LFCI values (Figure 4). The mean LTS and MTD were significantly greater in the valgus ACL group compared with the TD reference group. Still, no significant differences were observed when comparing the valgus ACL group with the non-valgus ACL group, or the non-valgus ACL group with the TD reference group (Supplemental Figure S2). The NWI and the MTS showed no significant mean differences between the TD group and the valgus and non-valgus ACL rupture groups (Table 3). The LTH was significantly lower in the TD group compared with both the valgus and non-valgus ACL rupture groups.

**Table 3:** Comparison of Bony Morphological Parameters Between Groups*^*a*^*

Parameter	TD Group (n = 26)	Valgus ACL Patients (n = 26)	Non-valgus ACL Patients (n = 26)	Valgus vs TD*^*b*^*	*P* *^*b*^*	Valgus vs Non-valgus*^*c*^*	*P* *^*c*^*	Non-valgus vs TD*^*d*^*	*P* *^*d*^*
LFCI	0.77 ± 0.05	0.64 ± 0.08	0.75 ± 0.09	−0.16 (–0.20 to −0.11)	**<.001**	−0.12 (–0.17 to −0.08)	**<.001**	−0.03 (–0.07 to 0.01)	.118
LTS, deg	5.35 ± 3.95	8.19 ± 3.68	6.65 ± 2.86	2.53 (0.50 to 4.56)	**.016**	1.23 (–0.82 to 3.28)	.195	1.30 (–0.68 to 3.28)	.195
LTH, mm	3.36 ± 0.70	4.01 ± 0.87	3.99 ± 0.63	0.68 (0.10 to 1.26)	**.002**	0.03 (–0.40 to 0.45)	.940	0.65 (0.24 to 1.06)	**.002**
MTS, deg	5.53 ± 2.57	5.72 ± 3.16	4.85 ± 3.02	−0.30 (–2 to 1.39)	.722	−0.23 (–1.48 to 1.94)	.792	−0.53 (–2.18 to 1.12)	.523
MTD, mm	1.40 ± 0.67	2.23 ± 1.01	1.92 ± 0.86	0.67 (0.20 to 1.15)	**.006**	0.25 (–0.23 to 0.72)	.308	0.43 (0.03 to 0.89)	.067
NWI	0.28 ± 0.04	0.27 ± 0.04	0.28 ± 0.04	−0.01 (–0.04 to 0.01)	.250	−0.01 (–0.04 to 0.01)	0.394	−0.00 (–0.03 to 0.02)	.764

a Data are presented as mean ± SD or AMD (95% CI). Bold *P* values indicate statistical significance. The valgus group: MAD <1 mm medial; the non-valgus group: MAD ≥1 mm medial. ACL, anterior cruciate ligament; AMD, adjusted mean difference; LFCI, lateral femoral condyle index; LTH, lateral tibial height; LTS, lateral posterior tibial slope angle; MAD, mechanical axis deviation; MTD, medial tibial depth; MTS, medial posterior tibial slope angle; NWI, notch width index; TD, typically developed control group.

b Valgus versus TD.

c Valgus versus non-valgus.

d Non-valgus versus TD.

ANCOVA, including all patients with ACL rupture (n = 150) without propensity score matching, demonstrated comparable results (Supplementary Table S1), providing a broader analysis that incorporated the full spectrum of variability in the cohort, avoided potential biases introduced by matching, and supported initial findings. Likewise, unadjusted analysis of variance, without the inclusion of age and sex covariates, also exhibited similar findings (Supplementary Table S2). Using more conservative alignment classifications, we found similar results; however, there were also significant differences in LFCI between the TD group and both alignment subgroups with ACL rupture (Table 4).

**Table 4:** Comparison of Bony Morphological Parameters Between Groups With Conservative Alignment Classifications*^*a*^*

Parameter	TD Group (n = 26)	Valgus ACL Patients (n = 26)	Non-Valgus ACL Patients (n = 26)	Valgus vs TD*^*b*^*	*P* *^*b*^*	Valgus vs Non-Valgus^*c*^	*P* *^*c*^*	Non-Valgus vs TD*^*d*^*	*P* *^*d*^*
LFCI	0.77 ± 0.05	0.65 ± 0.07	0.71 ± 0.10	−0.14 (–0.20 to −0.08)	**<.001**	−0.07 (–0.12 to −0.01)	**.020**	−0.07 (–0.12 to −0.03)	**.002**
LTS, deg	5.35 ± 3.95	8.13 ± 4.02	7.11 ± 3.04	2.78 (0.09 to 5.48)	**.041**	0.69 (–1.66 to 4.82)	.561	1.70 (–0.16 to 3.55)	.072
LTH, mm	3.36 ± 0.70	3.79 ± 0.86	4.09 ± 0.69	0.45 (–0.04 to 0.95)	.072	−0.29 (–0.77 to 0.19)	.226	0.74 (0.37 to 1.12)	**<.001**
MTS, deg	5.53 ± 2.57	5.76 ± 3.59	5.08 ± 2.89	−0.35 (–2.35 to 1.66)	.733	0.11 (–1.83 to 2.04)	.912	−0.45 (–1.98 to 1.08)	.558
MTD, mm	1.40 ± 0.67	2.16 ± 1.14	2.03 ± 0.86	0.53 (0.03 to 1.04)	.064	0.24 (–0.56 to 0.52)	.949	0.55 (0.12 to 0.98)	**-.013**
NWI	0.28 ± 0.04	0.27 ± 0.04	0.28 ± 0.04	−0.01 (–0.04, 0.02)	0.457	−0 (–0.03 to 0.03)	.824	−0.01 (–0.03 to 0.01)	.824

a Data are presented as mean ± SD or AMD (95% CI). Bold *P* values indicate statistical significance. The conservative alignment classification of MAD^15^ is composed of valgus groups (MAD ≥10 mm lateral) and non-valgus groups (MAD <10 mm lateral).

ACL, anterior cruciate ligament; AMD, adjusted mean difference; LFCI, lateral femoral condyle index; LTH, lateral tibial height; LTS, lateral posterior tibial slope angle; MAD, mechanical axis deviation; MTD, medial tibial depth; MTS, medial posterior tibial slope angle; NWI, notch width index; TD, typically developed control group.

b Valgus versus TD.

c Valgus versus non-valgus.

d Non-valgus versus TD.

Regression analyses demonstrated that age was weakly correlated with LFCI (*r* = 0.356; *P* < .001), but no other morphological parameter (Supplementary Table S3 and Figures S3 and S4). Given that our primary analysis was matched for age and sex, these exploratory findings would not have significantly altered the reported mean differences.

Alignment measures (MAD, mLDFA, and MPTA) were not collected for the TD group because of the absence of a clinical indication for long-leg alignment films. Medial and lateral mechanical axis deviations are labeled explicitly for clarity—The valgus group (MAD <1 mm medial); the non-valgus group (MAD ≥1 mm medial).

## Discussion

Our study demonstrates that distinct morphological variations exist within pediatric patients presenting with ACL rupture, which may contribute to differences in lower-limb alignment. Valgus-aligned ACL patients had smaller lateral femoral condyles, as reflected by lower mean LFCI values compared with both non-valgus ACL patients and the TD group (Table 3). Moreover, valgus-aligned ACL patients exhibited steeper mean lateral tibial slopes compared with the TD group. These differences in lateral compartment morphology highlight how smaller femoral condyles and steeper posterior tibial slopes may predispose valgus-aligned individuals to dynamic instability and ACL rupture. Collectively, our findings underscore the morphological distinctions between the TD, valgus ACL, and non-valgus ACL groups. To our knowledge, our study is the first to investigate knee morphology and its relationship to alignment in a pediatric population with ACL injuries. As anatomic changes occur throughout growth, future research should incorporate longitudinal assessments of bony morphology to better understand its role in injury risk.

Our findings demonstrated that LFCI values were significantly smaller in valgus-aligned ACL patients compared with both the non-valgus ACL group and the TD group, supporting our hypothesis that altered lateral femoral condylar morphology is associated with ACL rupture. Given that the LFCI is a ratio derived from the flexion and extension curvatures of the lateral femoral condyle, even small changes in this ratio may reflect meaningful differences in condylar geometry, which can affect knee joint biomechanics. A recent study of adult athletes with ACL rupture found no significant differences in LFCI when comparing their uninjured knees to a healthy control group,^17^ suggesting that the LFCI may not have the clinical value initially assumed. Nowak et al^17^ cited limitations within the measurement methodology of the LFCI, particularly in knees without a distinct notch to clearly define the anterior and posterior extension circles. In such ambiguous cases, variability and randomness in LFCI measurements were observed, potentially limiting the interpretability of this parameter. However, the random distribution of measurement variability favors the robustness of the LFCI, as such variability would not influence the directional relationship between LFCI and ACL rupture. Furthermore, intra- and interrater reliability^8,17^ of the LFCI have been documented to be excellent, consistent with our study's findings. To account for potential geometric variability and slice differences in LFCI measurements, we adopted a conservative approach to minimize bias and prevent overestimation of group differences (type 1 error). Nowak et al^17^ also compared patients’ uninjured knees with healthy controls, highlighting how trauma and ACL rupture can alter joint morphology and flatten the femoral condyle, potentially limiting the investigation of the injured limb's LFCI. Notably, a computed tomography study of adult knees found that only 26% of patients exhibited identical alignment phenotypes,^21^ potentially suggesting symmetry of adult knees is an uncommon phenomenon. Given that asymmetric alignment has been observed in previous registry-based studies^22^ of patients with pediatric ACL rupture, symmetry of morphology in the contralateral limb cannot be assumed. Therefore, the interpretation of an uninjured knee's LFCI may be limited. In contrast, our study focused on the injured limb, with MRI performed at a median of 17 days after injury, minimizing the likelihood of growth- or injury-related changes. Although bony morphology can evolve in individuals with open growth plates, this short interval supports the validity of our findings. Likewise, age and sex matching accounted for growth variability in statistical analysis, and no meaningful exploratory correlations were identified between age and morphology. Our conservative alignment analysis also showed similar results to our initial findings, highlighting the repeatability of the LFCI and other measures. Therefore, our study accounted for growth- and injury-related variability unique to the pediatric population.

Our study's findings are associations, and we cannot conclusively state that these morphological factors directly cause ACL injury. However, we believe that these morphological parameters reflect intrinsic preinjury bony structures and are unlikely to be significantly influenced by acute injury, effusion, or muscle guarding. Altered femoral morphology may exaggerate the pivoting mechanism of the knee at the time of ACL rupture.^8^ A smaller LFCI may increase gliding over the lateral tibial plateau because of a disproportionately large anterior extension circle relative to the posterior extension circle (see Figure 2). On the tibial side, increased lateral tibial slope has been documented to decrease anteroposterior stability, leading to greater anterior tibial translation and subsequent strain through the ACL.^9^ Such tibiofemoral asymmetry in the lateral compartment may alter joint contact forces in the knee. Potentially, a hypoplastic lateral femoral condyle or steeper lateral tibial slope may be reflected by coronal plane valgus alignment. This alteration of bony structures may influence the anatomic station of the knee and, therefore, alignment. Indeed, dynamic valgus moments^18^ predispose to ACL rupture. However, there is a paucity of evidence exploring the relationship between static and dynamic alignment. Given that ACL injuries are a result of a complex combination of forces, primarily dynamic valgus alongside internal rotation of the knee, underlying alteration of morphology may accentuate such forces. Therefore, decreased LFCI and static malalignment may exist on a continuum to predispose a patient to ACL rupture. However, it is important to note that morphological parameters are static measures that may have only tenuous contributions to the dynamic biomechanics of an ACL rupture.

### Clinical Implications

In adult populations,^8^ smaller LFCI values have been associated with increased risk of ACL rupture and graft failure, which underscores the need to evaluate whether these findings translate to the pediatric population. Altered joint loading in the lateral compartment owing to the LFCI or malalignment may predispose patients to long-term sequelae, such as early osteoarthritis, secondary meniscal tears, and graft failure. Understanding this association may inform clinical decision-making, particularly in identifying high-risk patients as well as guiding the use of adjunctive procedures. For instance, pediatric patients with decreased LFCI undergoing ACL reconstruction may theoretically benefit from extra-articular stabilizing procedures to address rotational instability. Similarly, if decreased LFCI contributes to coronal plane malalignment, a hypothetical realignment osteotomy or hemiepiphysiodesis^5^ performed alongside ACL reconstruction may not fully address the underlying 3-dimensional morphological abnormalities represented by the LFCI. Because the LFCI reflects overall condylar sphericity, single-plane coronal corrections alone may be insufficient to restore optimal joint biomechanics, potentially leaving patients vulnerable to further instability or degenerative changes. However, additional research is warranted to assess how the LFCI influences ACL injury risk, surgical outcomes, and long-term joint health. Specifically, growth-related changes to pediatric knee morphology should be investigated to better understand injury susceptibility and guide subsequent treatment considerations.

### Limitations

This study has several limitations. First, varus (MAD ≥15 mm medial) and neutral-aligned patients were combined into 1 group because of the small number of varus patients (n = 6) within the eligible registry cohort (n = 150). Despite this, conservative alignment classifications produced similar results, confirming the robustness of our findings. Second, there was an imbalance between valgus and non-valgus alignment groups, which we addressed through propensity score matching for age and sex, ensuring adequately powered groups of equal sample size. Supplementary analysis, including all patients, irrespective of group size, yielded consistent results (Supplementary Table S2). Third, long-leg alignment films were not collected for the healthy TD cohort, as there was no clinical indication for radiographic imaging. Our study's focus was on establishing baseline morphological characteristics in TD children, and we do not anticipate that minor alignment variations in this healthy control group would significantly affect comparisons of morphology between the injured and uninjured groups. Fourth, the timing of imaging at a median of 17 days after injury could theoretically introduce minor confounding because of acute injury-related changes or growth effects. However, this short interval and the inclusion of age as a covariate in the statistical models mitigated these concerns. Finally, while measurement error is an inherent limitation when comparing mean differences, inter- and intrarater reliability metrics demonstrated strong agreement, indicating a low margin of error and supporting the robustness of our measurements.

## Conclusion

Our study demonstrates that morphological variations—including altered lateral femoral condyles and tibial morphology—are associated with ACL rupture in pediatric patients, particularly when accounting for lower limb alignment. Specifically, decreased LFCI and increased LTS were associated with valgus-aligned patients. Further research is required to determine how altered knee-joint morphology influences both the risk of ACL rupture and subsequent clinical outcomes.

## Supplemental Material

sj-pdf-1-ojs-10.1177_23259671251365625 – Supplemental material for Variable Knee-Joint Morphology In Valgus and Non-valgus Aligned Pediatric Patients With Anterior Cruciate Ligament Rupture Supplemental material, sj-pdf-1-ojs-10.1177_23259671251365625 for Variable Knee-Joint Morphology In Valgus and Non-valgus Aligned Pediatric Patients With Anterior Cruciate Ligament Rupture by Alexander H. Seeto, Kylie Bradford, David Bade, Liam Johnson, Sheanna Maine, David J. Saxby, Christopher P. Carty and Ivan P. Astori in Orthopaedic Journal of Sports Medicine
