# Supplementary material for: Variable Knee-Joint Morphology In Valgus and Non-valgus Aligned Pediatric Patients With Anterior Cruciate Ligament Rupture
Source: Orthop J Sports Med. 2025 Sep 25;13(9):23259671251365625. doi: 10.1177/23259671251365625 (PMC12464393; doi:10.1177/23259671251365625)

SUPPLEMENTAL MATERIAL

Supplementary Table 1: Comparison of bony morphological parameters of the entire cohort (ACL injured patients n=150, controls n=26), prior to propensity score matching

Parameter	Typically Developed (TD) Group (n=26, mean ± SD)	Valgus ACL Patients (n=112, mean ± SD)	Non-Valgus ACL Patients (n=38, mean ± SD)	Valgus vs. TD[a] (Adjusted Mean Difference, 95%CI)	p-value[a]	Valgus vs. Non-Valgus[b] (Adjusted Mean Difference, 95%CI)	p-value[b]	Non-Valgus vs. TD[c] (Adjusted Mean Difference, 95%CI)	p-value[c]
LFCI	0.77 ± 0.05	0.63 ± 0.07	0.75 ± 0.09	-0.10 (-0.14, -0.05)	**<0.001**	-0.09 (-0.13, -0.05)	**<0.001**	-0.00 (-0.06, 0.05)	0.979
LTS [°]	5.35 ± 3.95	8.08 ± 3.50	6.74 ± 2.90	2.01 (0.49, 4.56)	**0.035**	0.65 (-0.95, 2.26)	0.602	1.00 (-1.29, 3.28)	0.560
LTCH [mm]	3.36 ± 0.70	4.08 ± 0.83	4.02 ± 0.63	0.31 (-0.09, 0.72)	0.159	0.06 (-0.44, 0.31)	0.681	0.43 (-0.03, 0.89)	0.076
MTS [°]	5.53 ± 2.57	5.72 ± 3.16	4.85 ± 3.02	-0.10 (-1.63, 1.41)	0.986	0.20 (-0.93, 1.48)	0.875	-0.36 (-2.11, 1.39)	0.878
MTD [mm]	1.40 ± 0.67	2.11 ± 0.91	1.95 ± 0.89	0.91 (0.42, 1.40)	**0.001**	0.03 (-0.36, 0.43)	0.894	0.88 (0.31, 1.44)	**0.001**
NWI	0.28 ± 0.04	0.27 ± 0.05	0.28 ± 0.04	-0.01 (-0.04, 0.01)	0.250	-0.01 (-0.04, 0.01)	0.394	-0.00 (-0.03, 0.02)	0.764

ACL = Anterior Cruciate Ligament, TD = Typically Developed Control Group. MAD = Mechanical Axis Deviation, LFCI = Lateral Femoral Condyle Index, LTS = Lateral Posterior Tibial Slope Angle, LTCH = Lateral Tibial Chondral Height, MTS = Medial Posterior Tibial Slope Angle, MTD = Medial Tibial Depth, NWI= Notch Width Index.

Valgus Group (MAD < 1mm medial), Non-Valgus Group (MAD ≥ 1 mm medial).

Values are parametric and are reported as mean ± standard deviation (SD).

Superscripts indicate group comparisons: a = Valgus vs. TD, b = Valgus vs. Non-Valgus, c = Non-Valgus vs. TD.

MD = Mean Difference; 95%CI= Confidence Interval, adjusted for age and sex. Bolded p-values indicate statistical significance.

Supplementary Table 2: Unadjusted comparison of bony morphological parameters between groups.

**Parameter**	**Typically Developed (TD) Group (n=26, mean ± SD)**	**Valgus ACL Patients (n=26, mean ± SD)**	**Non-Valgus ACL Patients (n=26, mean ± SD)**	**Valgus vs. TD[a] (Adjusted Mean Difference, 95%CI)**	**p-value[a]**	**Valgus vs. Non-Valgus[b] (Adjusted Mean Difference, 95%CI)**	**p-value[b]**	**Non-Valgus vs. TD[c] (Adjusted Mean Difference, 95%CI)**	**p-value[c]**
LFCI	0.77 ± 0.05	0.64 ± 0.08	0.75 ± 0.09	-0.14 (-0.18, -0.09)	**<0.001**	-0.11 (-0.16, -0.06)	**<0.001**	-0.03 (-0.08, 0.02)	0.376
LTS [°]	5.35 ± 3.95	8.19 ± 3.68	6.65 ± 2.86	2.84 (0.54, 5.15)	**0.012**	1.54 (-0.73, 3.80)	0.243	1.31 (-0.98, 3.59)	0.364
LTCH [mm]	3.36 ± 0.70	4.01 ± 0.87	3.99 ± 0.63	0.65 (0.17, 1.14)	**0.007**	0.07 (-0.45, 0.59)	0.701	0.63 (0.15, 1.11)	**0.007**
MTS [°]	5.53 ± 2.57	5.72 ± 3.16	4.85 ± 3.02	-0.19 (-1.72, 1.12)	0.968	0.88 (-1.01, 2.77)	0.511	-0.68 (-2.59, 1.23)	0.671
MTD [mm]	1.40 ± 0.67	2.23 ± 1.01	1.92 ± 0.86	0.83 (0.26, 1.39)	**0.002**	0.31 (-0.24, 0.87)	0.376	0.51 (-0.05, 1.07)	0.079
NWI	0.28 ± 0.04	0.27 ± 0.04	0.28 ± 0.04	-0.01 (-0.04, 0.01)	0.475	-0.01 (-0.04, 0.01)	0.376	-0.00 (-0.02, 0.03)	0.988

ACL = Anterior Cruciate Ligament, TD = Typically Developed Control Group. MAD = Mechanical Axis Deviation, LFCI = Lateral Femoral Condyle Index, LTS = Lateral Posterior Tibial Slope Angle, LTCH = Lateral Tibial Chondral Height, MTS = Medial Posterior Tibial Slope Angle, MTD = Medial Tibial Depth, NWI= Notch Width Index.

Valgus Group (MAD < 1mm medial), Non-Valgus Group (MAD ≥ 1 mm medial).

Values are parametric and are reported as mean ± standard deviation (SD).

Superscripts indicate group comparisons: a = Valgus vs. TD, b = Valgus vs. Non-Valgus, c = Non-Valgus vs. TD.

MD = Mean Difference; 95%CI= Confidence Interval, adjusted for age and sex. Bolded p-values indicate statistical significance.

Supplementary Figure 1: Supplemental Measurement Protocol

Reference Line for Lateral Tibial Slope (LTS) and Medial Tibial Slope (MTS)

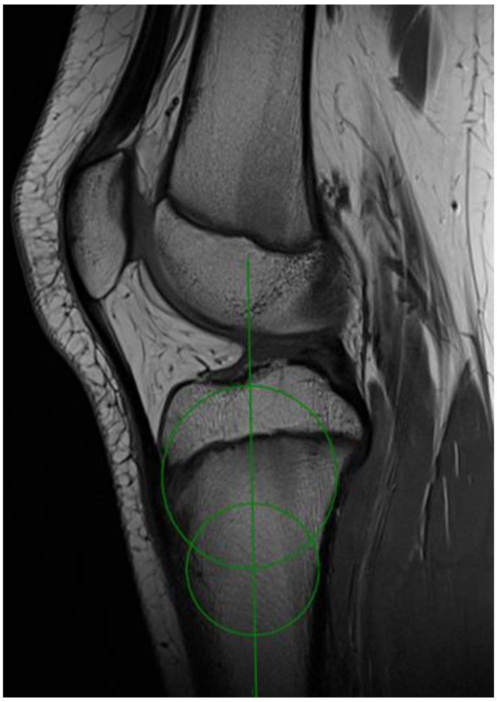

Lateral Tibial Slope (LTS)

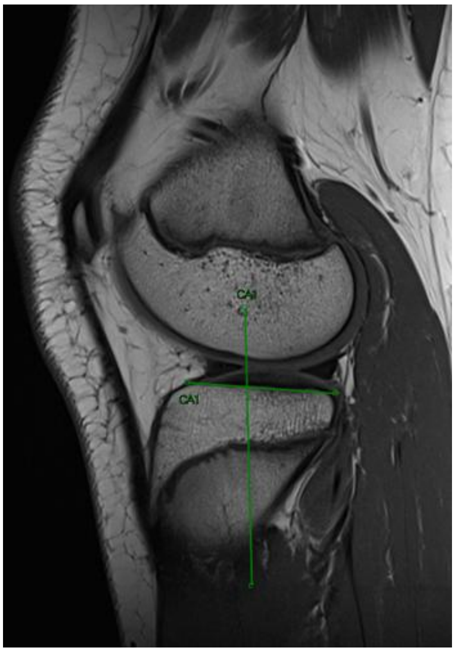

Lateral Tibial Chondral Height (LTCH)

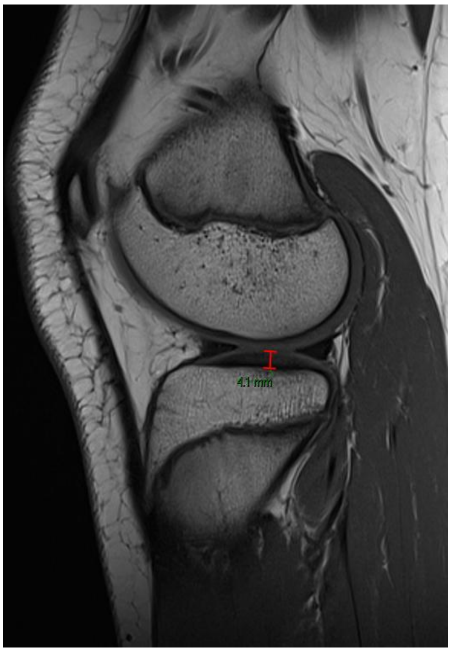

**Medial Tibial Slope (MTS)**	**Medial Tibial Depth (MTD)**
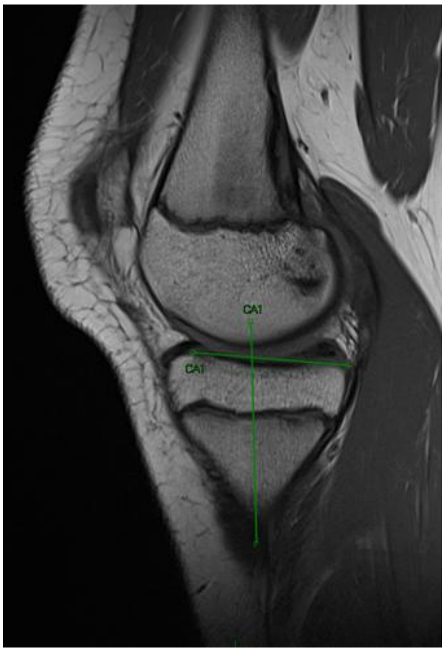	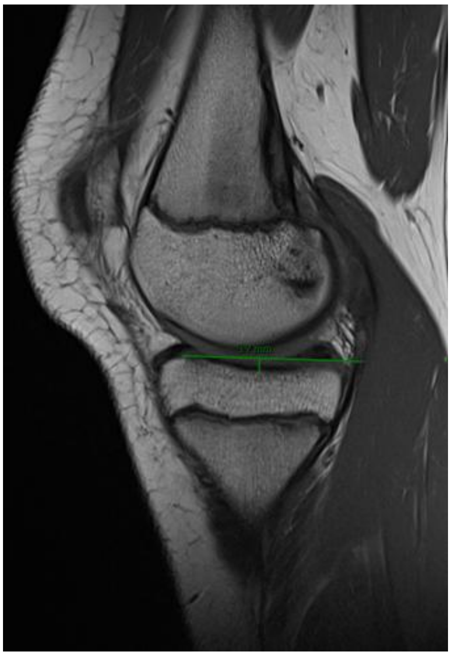
**Notch Width Index (NWI)**	
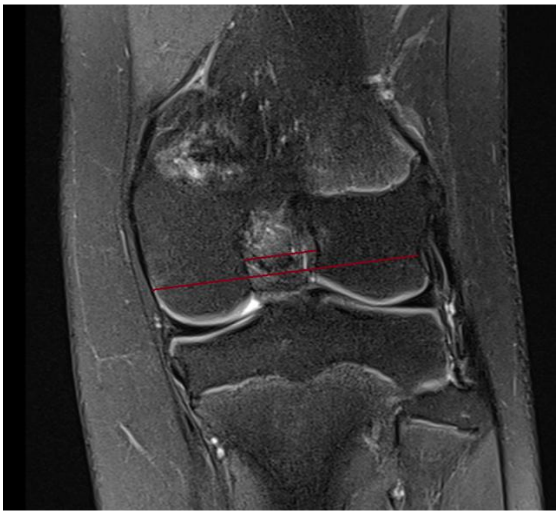	

Supplemental Figure 2: Box Plots of Other Morphological Parameters by Group

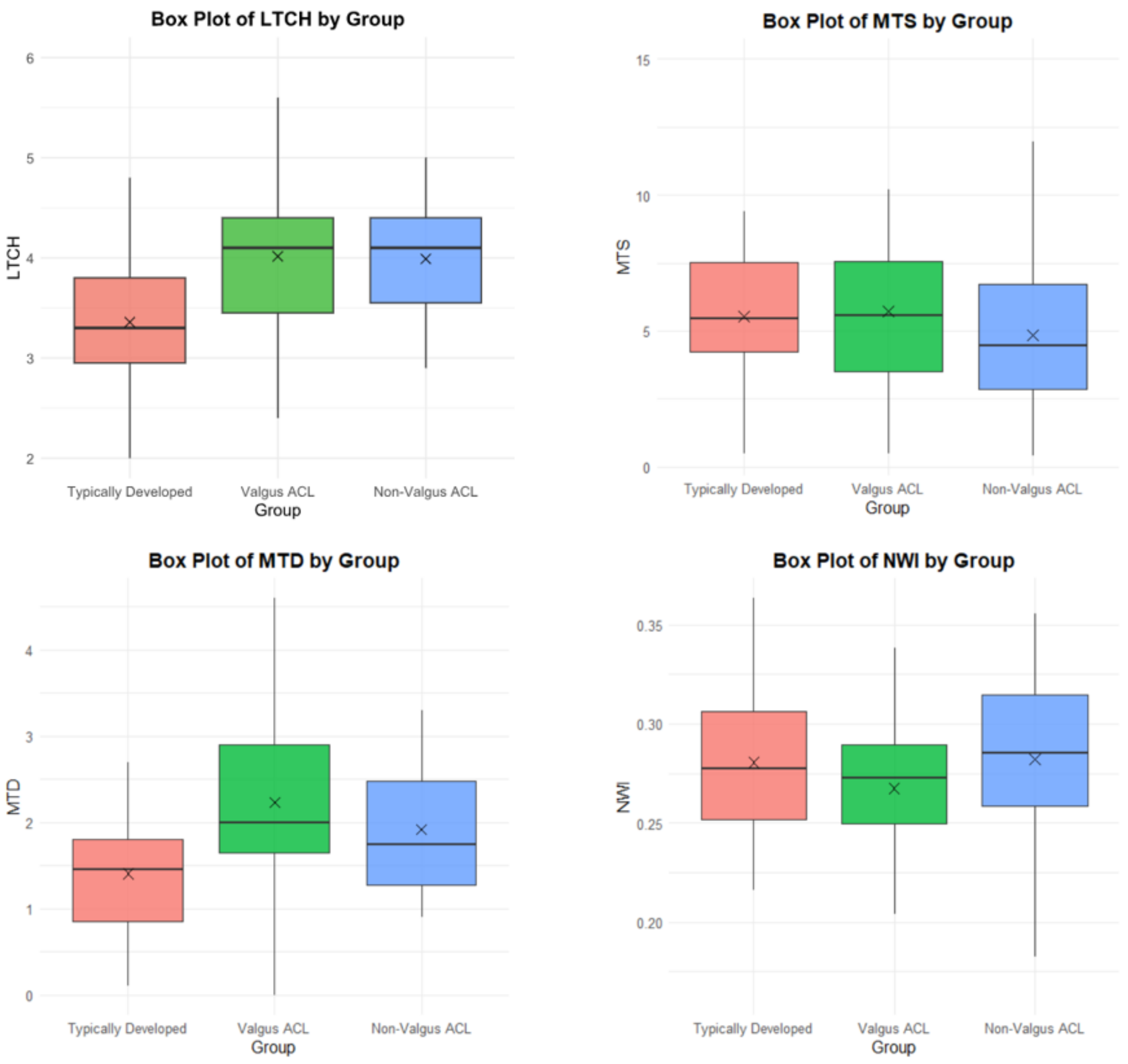

ACL = Anterior Cruciate Ligament, TD = Typically Developed Control Group. LTCH = Lateral Tibial Chondral Height (mm), MTS = Medial Posterior Tibial Slope Angle°, MTD = Medial Tibial Depth (mm), NWI= Notch Width Index.

Valgus Group (MAD < 1mm medial), Non-Valgus Group (MAD ≥ 1 mm medial). Black crosses represent group means.

<u>Age Analysis</u>

Supplementary Table 3: Correlation Analysis of Age and Morphological Parameters

Parameter	LFCI	LTS	LTCH	MTS	MTD	NWI
Correlation Coefficient	.356*	.048	.161	.082	.167	.153
P- Value	**.000**	.565	.051	.321	.053	.064

LFCI = Lateral Femoral Condyle Index, LTS = Lateral Posterior Tibial Slope Angle, LTCH = Lateral Tibial Chondral Height, MTS = Medial Posterior Tibial Slope Angle, MTD = Medial Tibial Depth, NWI= Notch Width Index.

Bolded *P* values indicate statistical significance.

* Analysis of LFCI and age in the valgus group (r = 0.400, *P* = 0.016), non valgus group (r = 0.313, *P* = 0.001), and TD group (r = 0.417, *P* = 0.053) yielded similar findings

Supplementary Figure 3: Scatter Plot of LFCI and Age

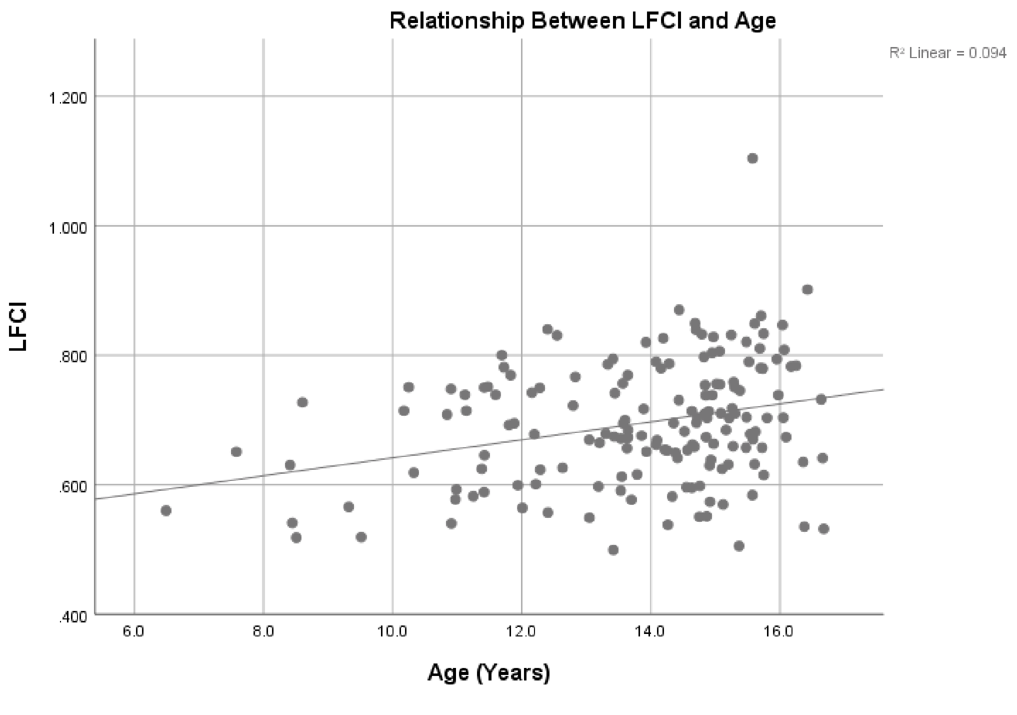

Given that the correlation coefficient was weak but statistically significant for lateral femoral condyle index (LFCI) and age (r = 0.356, $p < 0.05$), this was explored further using a scatter plot with a fitted regression line, demonstrating a weak positive association. The regression line shows that LFCI slightly increases with age, suggesting that older patients may have a larger lateral femoral condyle index. The R^2 value of 0.094 indicates that approximately 9.4% of the variance in LFCI is explained by age, implying that while there is a trend, most of the variability in LFCI is attributable to other factors. This supports the weak but statistically significant correlation coefficient.

Supplementary Figure 4: Histogram of Age Distribution

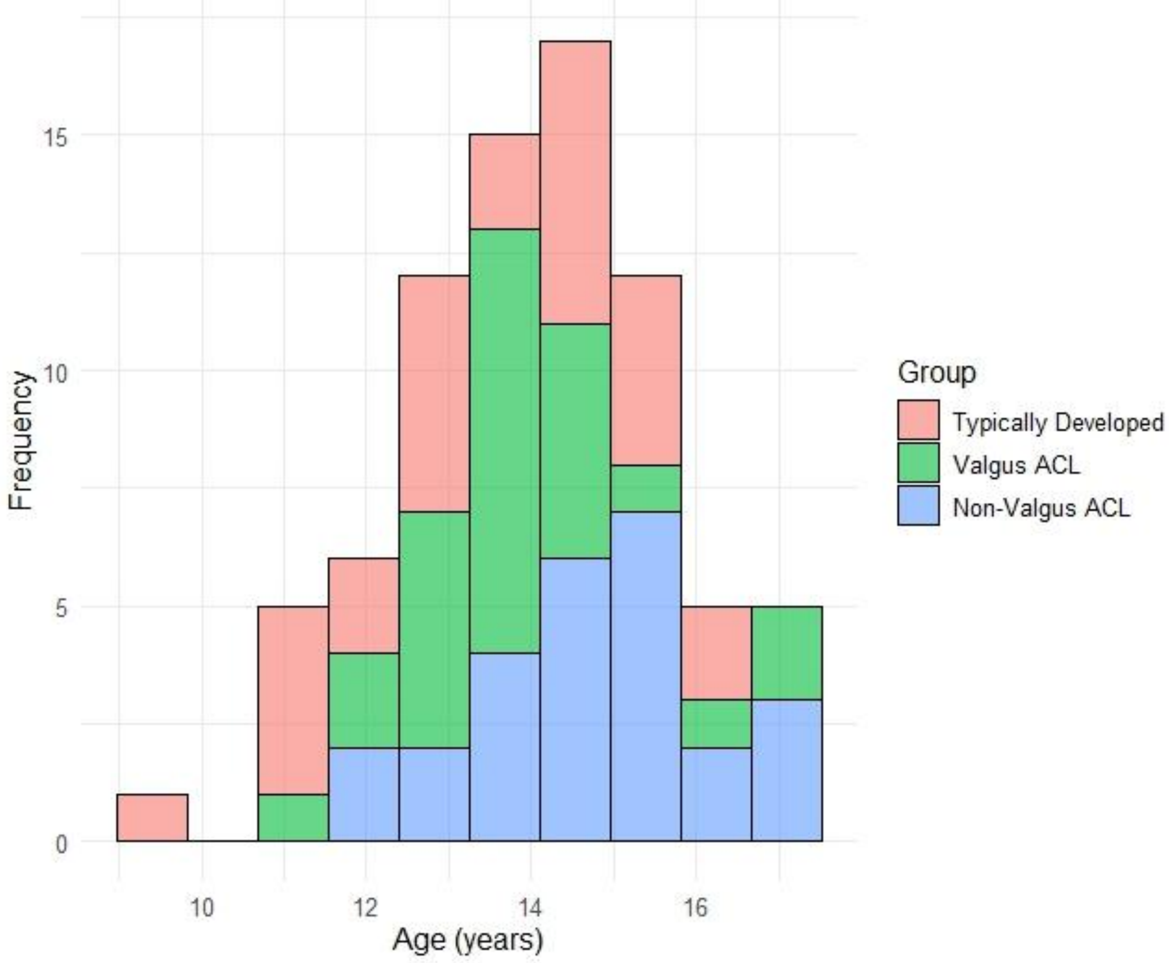

Supplement: sj-pdf-1-ojs-10.1177_23259671251365625 – Supplemental material for Variable Knee-Joint Morphology In Valgus and Non-valgus Aligned Pediatric Patients With Anterior Cruciate Ligament Rupture [file sj-pdf-1-ojs-10.1177_23259671251365625.pdf]
